# Differential response of the soil nutrients, soil bacterial community structure and metabolic functions to different risk areas in Lead-Zine tailings

**DOI:** 10.3389/fmicb.2023.1131770

**Published:** 2023-09-12

**Authors:** Zexun Liu, Jiayao Zhuang, Kang Zheng, Chengcheng Luo

**Affiliations:** Collaborative Innovation Center of Sustainable Forestry in Southern China of Jiangsu Province, Nanjing Forestry University, Nanjing, China

**Keywords:** tailings, high-throughput sequencing, soil nutrients, bacterial community structure, metabolic function

## Abstract

Rapid growth in the mining industry has brought about a large formation of tailings, which result in serious destruction of the ecological environment and severe soil pollution problems. This study assesses soil nutrients, soil bacterial community and soil microbes’ metabolic function in heavily polluted areas (W1), moderately polluted areas (W2), lightly polluted areas (W3) and clean areas (CK) using 16S Illumina sequencing. The results of this study showed that compared with CK, a severe loss of soil nutrients and richness of OTUs (Chao1 and ACE indices) were observed with the aggravated pollution of tailings. The Chao1 and ACE indices in the W1 group decreased significantly by 15.53 and 16.03%, respectively, (*p* < 0.01). Besides, the relative abundance of *Actinobacteria* and *Proteobacteria* was high whereas and relative abundance of *Chloroflexi* in the polluted areas. Among them, W1 groups increased significantly the relative abundance of *Actinobacteria* and decreased significantly the relative abundance of *Chloroflexi*, these can be used as indicator phyla for changes in soil community structures under polluted stress. Tax4 Fun analysis showed that W1 groups affected the soil bacterial community and altered the primary types of biological metabolism in polluted areas. Tailings have adverse impacts on soil bacterial community and metabolic functions, and the deterioration in soil quality is dependent on the levels of tailings pollution. Cumulatively, this study provides valuable information on the bacterial community structure and metabolic functions in the tailing polluted soil.

## 1. Introduction

Microbes are vital to biogeochemical cycles necessary for healthy ecosystems (Madsen, 2011; Smith et al., 2015). As the most abundant and diverse form of life on Earth, changes in microbial communities provide insight into the health and functioning of complex environments (Whitman et al., 1998; Horner-Devine et al., 2004). Heavy-metal contamination has been implicated frequently in altering soil microbial community structure by reducing soil nutrient. Various factors, like soil pH, available phosphorous, and hydrolyzed nitrogen, may influence soil bacterial diversity (Li H. et al., 2021; Sheng et al., 2021). Moreover, it has also been documented that microbes show potential capacity in the remediation of heavy metals pollution in soil (Chen et al., 2020; Xie et al., 2021). some microbes have certain tolerance and remediation potential to harmful heavy metals due to long-term exposure and living in heavy metal contaminated soil, which directly weakens the toxic effect of heavy metals on soil (Zhang et al., 2011; Tamariz-Angeles et al., 2021). Besides, some microbes from plant roots exudates or inside plant tissues, including roots, stems and leaves, could have positive effects on promoting plant growth, thus improving the ability of plants to repair heavy metal pollutants in soil (Pang et al., 2021; Ma et al., 2022). It is generally known that soil pollution by heavy metals can affect soil microbial diversity and community structure directly or indirectly by changes the soil nutrient level (Zhang et al., 2019). The variation in soil microbial population reflects the dynamics of nutrient cycling and soil health (Zheng et al., 2022). Hence, it is imperative to figure out how the soil quality and bacterial structure changes in response to varying pollution levels of heavy metals in soil.

Previous studies have showed that soil microbial diversity and soil microbial community composition vary under different pollution degree of heavy metals in soil. For example, the relative abundance of soil *Actinomycetes* decreased significantly under heavy metal lead stress, and the decrease of *Actinomycetes* abundance could results from an increase in the lead pollution degree in soil (Wang et al., 2021). Besides, Li D. R. et al. (2021) reported that artificial simulated soil heavy metal stress significantly reduced the diversity of soil bacterial community, while a little microbes could adapt to environmental changes result in increase their relative abundance. So far, various studies were mainly focused on the changes in soil bacterial diversity and bacterial community structure in mining areas (He et al., 2017; Maillet et al., 2020; Yang et al., 2022). However, few studies have explicitly focused on how different pollution levels of heavy metal of soil could affect the soil bacterial diversity, bacterial community structure and metabolic functions, especially in the tailings area. Therefore, understanding the underlying differences of soil bacterial diversity, bacterial community structure and metabolic functions under different pollution levels of heavy metals in the tailings is of great interest.

The lead-zinc mines in China are widely distributed and abundant in reserves (Liang et al., 2011), and lead and zinc are also momentous targets for heavy metal pollution remediation (Ma et al., 2023). Tailings are mainly used to store the wastes left by mining and are generally complex and diverse. With the rapid development of industrialization, mining has resulted in high tailing accumulation and occupied considerable land resources. It has also adversely affected the surrounding ecological environment (Mendez and Maier, 2008; Huang et al., 2021). Besides, tailings that contain a large amount of heavy metals lead and zinc, can easily pollute the soil, which would in turn potentially threaten human health (Timothy et al., 2019; Wang, 2019; Perrett et al., 2021). Besides, due to long-term mining and unreasonable management, the tailings ponds formed by lead-zinc mining activities have been accumulating for years, forming a continuous source of heavy metal pollution, resulting in a serious decline in soil quality and possible changes in soil microbial diversity and community structure. Although studies on the soil remediation of the lead-zinc mining areas have been carried out for decades, an effective treatment for the polluted soil surrounded by zinc mining tailings still warrant investigation (Carpenter et al., 2015; Zai, 2021). Hence, it is of great significance to understand the potential differences in bacterial communities in lead-zinc tailings and surrounding soils.

In this study, we evaluate the pollution levels of different tailings areas by comprehensive analyzed the contents of heavy metals lead and zinc in the soil using Nemera comprehensive pollution index method (*P*_N_). Besides, we also used high-throughput Illumina MiSeq sequencing technology to investigate the bacterial community structures under different polluted areas of tailings. This study aimed to determine (a) the changes of soil nutrients, soil bacterial diversity, bacterial community structure and metabolic function under different polluted areas of tailings, (b) the environmental factors influence the bacterial community composition, and (c) the key species of bacterial communities residing in tailings. These research results could provide basic data and theoretical support for tailings restoration and sustainable management.

## 2. Materials and methods

### 2.1. Experimental area and experimental design

**Figure 1:**
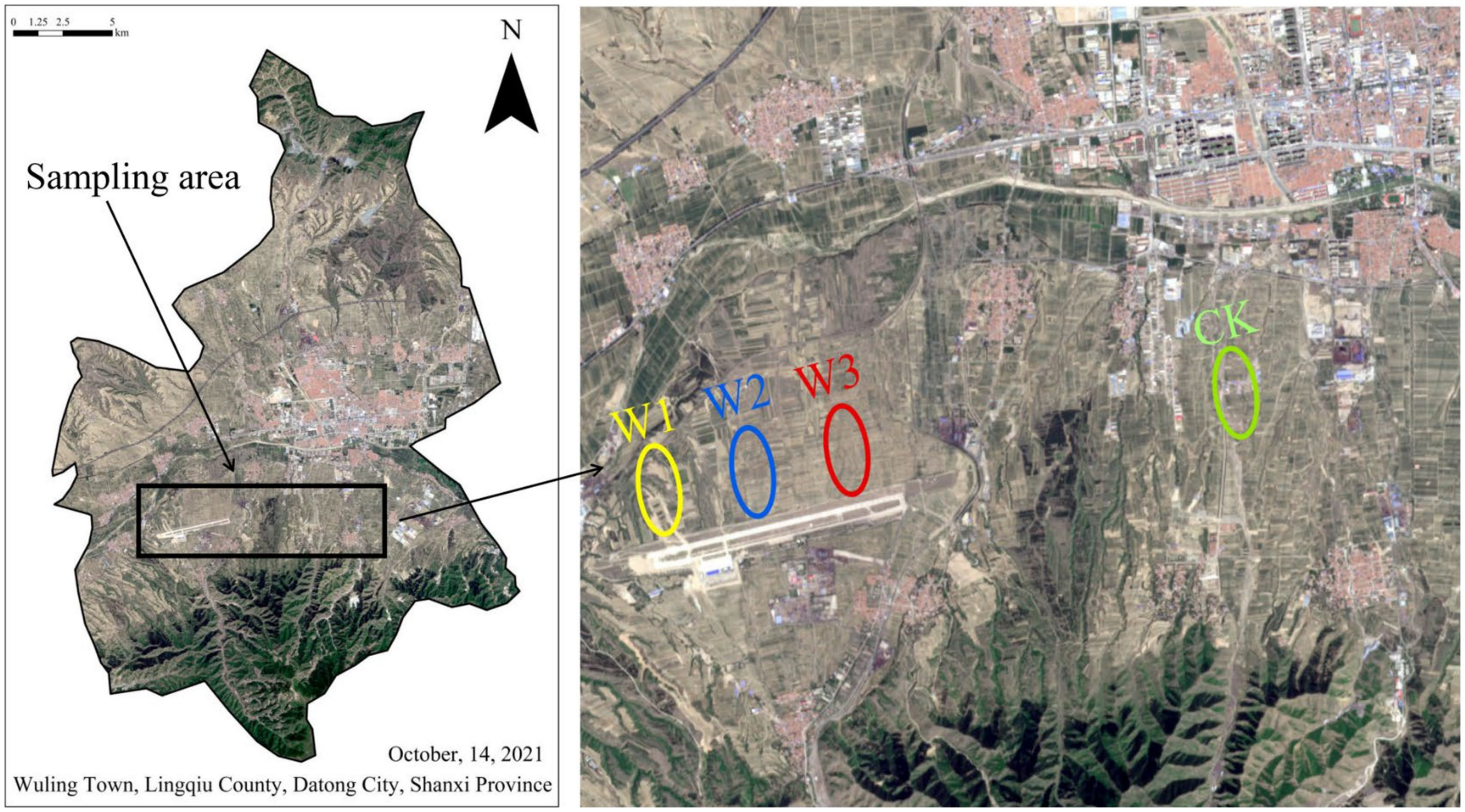
Remote sensing image map of the study area, and location of four sample plots.

ZhiJiadi lead-zinc mine is located at Gaojiazhuang Township(114°12′41″E, 39°21′30″N)in the South of Lingqiu County, Shanxi Province, China (Figure 1). This area has a temperate monsoon continental climate, the average annual temperature is 7°C, the annual precipitation is 432.4 mm, and the frost-free period is about 145 days on average. The tailings dam is located at the top of the hillside, and there is a road from the top of the tailings to the bottom. In this study, tailings were used as the pollution source, and four sample plots along the tailings dam and slopes were selected as the research objects, namely W1, W2, W3, and CK. W1 is located on the tailings slope, with no vegetation cover, mainly physical protection; W2 is located on the tailings slope, and the main vegetation is annual herbs; Located at the low slope of W3 tailings, the vegetation type is 15a poplar; The land use type of CK is farmland, adjacent to W3, and the crop type is corn. Due to the influence of tailings, the risk degree of heavy metal pollution was assessed for each sample plot.

### 2.2. Soil sampling time

**Figure 2:**
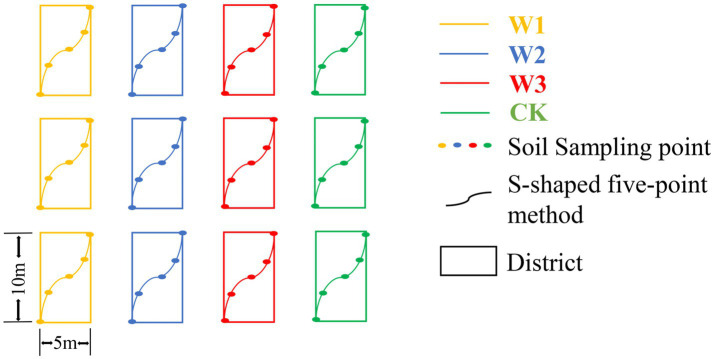
Layout of sample districts.

The soil sampling was carried out in October 2021. The sampling area was divided into 12 districts, with a district of 5 m × 10 m, and the soil samples from 0 to 20 cm depth were collected from each district using the S-shaped five-point mixed sampling method with a root drill with an inner diameter of 7 cm in each district. The soil from each district was mixed as a sample, so 12 samples were collected (Figure 2). The soil samples were stored in the self-sealing bag. The selected soil samples were divided into two parts and stored. One portion of the soil was stored in a 4°Cice box and brought back to the laboratory for further analysis. The second portion of the soil samples was air-dried to determine the physical and chemical properties of the soil.

### 2.3. Soil DNA extraction, PCR amplification, and high-throughput gene sequencing

The instructions of Mo Bio Powersoil® DNA Isolation Kit (Mo Bio Carlsbad CA, United States) were followed to extract total genomic DNA from each 0.25 g soil sample. To decrease the influence of soil heterogeneity on the test results and avoid the deviation of single DNA extraction and the influence of low DNA content in the samples on the test results, DNA from each of 12 soil samples was extracted several times and then pooled for subsequent analysis. The purity, concentration, and integrity of DNA samples were determined using NanoDrop 1,000 spectrophotometer (Thermo Scientific, DE, United States), Picogreen method (Quan-tus, PA, United States), and agarose gel electrophoresis method (Luo et al., 2023). After the test, the extracted genomic DNA was used as template, and 338F (5′-ACTCCTACGGGAGGCAGCAG-3′) and 806R (5′-GGACTAC HVGGGTWTCTAAT-3′) were used for PCR amplification and Illumina HiSeq sequencing of V3-V4 region of bacterial 16S rRNA gene (Xu et al., 2016). The PCR amplification steps are shown in Table 1. The PCR products were tested by electrophoresis with 2% agarose gel. The qualified products were purified by magnetic beads and quantified by enzyme labeling. According to the concentration of PCR products, the same amount of samples were mixed. After mixing, the PCR products were tested by electrophoresis with 2% agarose gel (Axygen BioSciences Inc., United States). The obtained target band was recovered by QIAquick Gel Extraction (Qiagen, #28704, Germany) kit. The recovered PCR product was stored in dry ice and sent to Guangzhou Kidio Science and Technology Service Company for sequencing by NovaSeq6000.

**Table 1:** 16S Polymerase chain reaction.

Reaction step		T (°C)	Time
Predenaturation		95	3 min
25 cycles	Denature	95	30s
	Anneal	55	30s
	Extend	72	45 s
Final extension		72	5 min

The PCR amplification steps were as follows: the predenaturation at 95°C for 3 min, followed by thirty-five cycles at 95°C for 30s, 55°C for 30 s, and 72°C for 45 s, were performed with a final extension at 72°C for 5 min. The total volume of the PCR reaction was 25 μ L.

### 2.4. Bioinformatics analysis

Bacterial 16S rRNA genes in the above-mentioned soil total genomic DNA samples were sequenced using the Illumina MiSeq platform of Kidio Science and Technology Service Company (Guangzhou, China). The genes were amplified with the primer sets 338F/806R (16S rDNA V3-V4 region gene), respectively (Xu et al., 2016). The sequence reads were assigned to each sample based on their unique barcode, and the original data obtained was quality filtering and chimera removal by using FLASH and trimmomatic software (Magoc and Salzberg, 2011; Li et al., 2022). Sequences with a sequence length of 50 bp, quality less than 20, and unclear bases were removed (Edgar et al., 2011) to obtain high-quality sequence dataset for subsequent information analysis. The filtered high-quality sequences with >97% similarity were assigned to the same OTU through USEARCH software (Edgar, 2010). The taxonomic identities of the bacteria were determined using RDP software (Wang et al., 2007) and Silva schemes (Quast et al., 2012).

When analyzing the number of OTUs in different polluted areas for making Venn diagram, our counting standard is as follows: When calculating the total number of OTUs in each polluted area, we take all the OTUs measured in three replicates in each polluted area as OTUs in each polluted area, although each repeated OTU does not appear in all replicates. If one OTU appears in two or three repetitions in each polluted area, in principle, it is regarded as the same OTU in the group, but the total number of OTUs in each group is not changed. When analyzing the number of overlapping OTUs between each polluted area, our calculation standard is that only when one OTU appears in three repetitions of each polluted area for no less than two times, will we regard it as an overlapping OTU.

### 2.5. Determination of the soil’s physical and chemical properties

The following indexes were determined in strict accordance with the experimental steps in the manual of the soil agrochemical analysis. Semi-micro Kjeldahl method was used to determine total nitrogen (TN) (Liu et al., 1996; Falco and Magni, 2004), the molybdenum-antimony anti-colorimetric method with NaOH melting was used to determine total phosphorus (TP), flame photometry with NaOH melting was used to determine total potassium (TK), alkaline hydrolysis diffusion method was used to determine hydrolyzed nitrogen (AN), molybdenum-antimony anti-colorimetric method with NaHCO_3_ extraction to determine available phosphorus (AP), flame photometry with NH_4_OAc extraction to determine available potassium (AK), and pH meter to determine pH, Soil organic carbon (OM) was determined by external heating with concentrated sulfuric acid and potassium dichromate. According to Wilson (2004), heavy metal contaminated soil was analyzed by digestion of 0.5 g of soil with 10 mL of concentrated HNO_3_, following the microwave-nitric acid method. The concentrations of Pb and Zn in the soil samples were measured using inductively Coupled Plasma Optical Emission Spectrometry (ICP-OES; Varian 710-ES).

### 2.6. Risk assessment of heavy metal pollution in soil

We comprehensive analyzed the contents of heavy metals lead and zinc to determined pollution levels of different soil samples by using Nemera comprehensive pollution index method (*P*_N_). the calculation formula is as follows:


$$P_{N}={\left\{\left[{\left(C_{i}/D_{i}\right)}^{2}{}_{\max}+{\left(C_{i}/D_{i}\right)}^{2}{}_{ave}\right]/2\right\}}^{1/2}$$

In the formula, *P*_N_ is the comprehensive pollution index value of soil heavy metals, *C*_i_ is the measured concentration value (mg·kg^−1^) of *i* soil heavy metals, and *D*_i_ is the soil background value (mg·kg^−1^) of Datong corresponding to *i* soil heavy metals (Cheng et al., 2014). According to the *P*_N_ value, the soil heavy metal pollution level can be divided into five grades, *P*_N_ ≤ 0.7 (clean), 0.7 < *P*_N_ ≤ 1 (warning value), 1 < *P*_N_ ≤ 2 (mild pollution), 2 < *P*_N_ ≤ 3 (moderate pollution) and *P*_N_ > 3 (severe pollution).

### 2.7. Statistical analysis

Statistical analysis of the soil’s physicochemical properties and contents of heavy metals lead and zinc in soil was carried out by applying one-way analysis of variance (ANOVA) and the new multiple range method to the data. All analyzes were conducted with SPSS statistical software package, version 20.0 (IBM, United States). Significance for statistical tests was accepted at *p* < 0.05.

The original sequence data and sequencing quality files were obtained from FASTQ files. The ACE and Chao indices was obtained to assess community alpha diversity by using Mothur software (Amato et al., 2013). The Unweighted-Unifrac algorithm in QIIME (v.1.8.0) software was used to analyze the hierarchical clustering and to examine PCoA of beta diversity. R’s default ggplot2 (version 4.2.1) package was used to make species composition analysis graphs. The correlations between the environmental factors, samples, and bacterial community were evaluated via redundancy analysis (RDA) using canoco software (version 4.5). Prior to conducting the RDA analysis, we standardized the units of physicochemical parameters using R’s default vegan package. This step ensured uniformity and consistency in the data. Tax4 Fun2 (version SILVA123) gene function prediction analysis builds a “species-gene” relationship network based on the complete genome. According to the annotation information of species in the SILVA123 database, OTUs are selected and classified. Then, it was standardized by the annotation information of NCBI genome, and the linear relationship between SILVA classification and original nuclear classification in the KEGG database is constructed to predict the gene function of test flora. All biological information analysis was carried out using the dynamic real-time interactive online data analysis platform.^1^

## 3. Results

### 3.1. Risk assessment of heavy metal pollution

The contents of Pb, Zn, and *P*_N_ in soils of different slope positions are shown in Table 2. According to the *P*_N_ value, W1, W2, W3 and CK groups are heavily polluted areas, moderately polluted areas, lightly polluted areas and clean areas, respectively.

**Table 2:** Risk assessment of heavy metal pollution in different slope positions of Datong tailings.

Index types	Background value	CK	W3	W2	W1
	(mg·kg^−1^)				
Pb	22.31	10.32 ± 1.03a ^2^	39.06 ± 2.12b	55.06 ± 0.98c	110.43 ± 2.34d
Zn	62.52	52.01 ± 6.26a	93.75 ± 4.33b	123.60 ± 5.12c	180.62 ± 5.26d
*P*_N_	*-*	0.68	1.82	2.23	4.45
Soil heavy metal pollution degree		Clean	Mild pollution	Moderate pollution	Severe pollution

CK, clean area; W1, heavily polluted area; W2, moderate pollution area; W3, slightly polluted area. Different lowercase letters (a, b, c, d) represent significant differences (*p* < 0.05) among heavily polluted areas (W1), moderately polluted areas (W2), lightly polluted areas (W3) and clean areas (CK).

### 3.2. Physical and chemical properties of the soil

**Figure 3:**
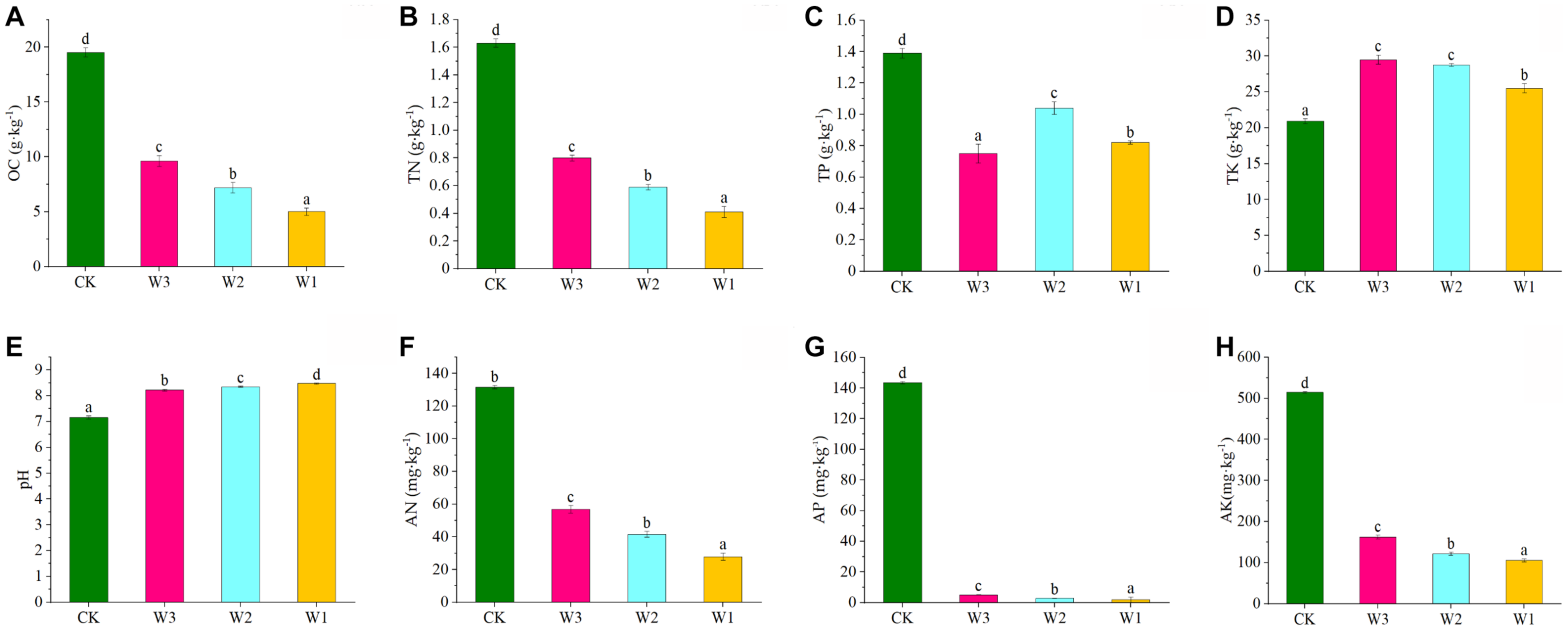
Variations in physical and chemical properties of soils under different polluted areas. **(A)** OC, organic matter, **(B)** TN, total nitrogen, **(C)** TP, total phosphorus, **(D)** TK, total potassium, **(E)** pH, **(F)** AN, available nitrogen, **(G)** AP, available phosphorus, **(H)** AK, available potassium. Here, Different lowercase letters indicate the significant differences (*p* < 0.05) among heavily polluted areas (W1), moderately polluted areas (W2), lightly polluted areas (W3) and clean areas (CK).

The Physical and chemical properties of the soil in the clean area and different polluted areas are detailed in Figure 3. The TN, AN, TP, AP, and OC levels were significantly decreased, and AK and pH levels were significantly increased in the soil from the polluted area (W1-W3) compared to the control (*p* < 0.05). At the same time, the contents of TN, AN, AP, and OC in the soil show gradually decreased with the aggravation of pollution degree. and in W1 decreased by 1.22 mg·kg^−1^, 103.73 mg·kg^−1^, 141.68 mg·kg^−1^, and 408.80 mg·kg^−1^, respectively with increasing pollution compared to CK. It showed that the soil area polluted by tailings had a significant impact on the accumulation of soil nutrients, resulting in the loss of soil nutrients.

### 3.3. Analysis of presence and absence of OTUs

**Figure 4:**
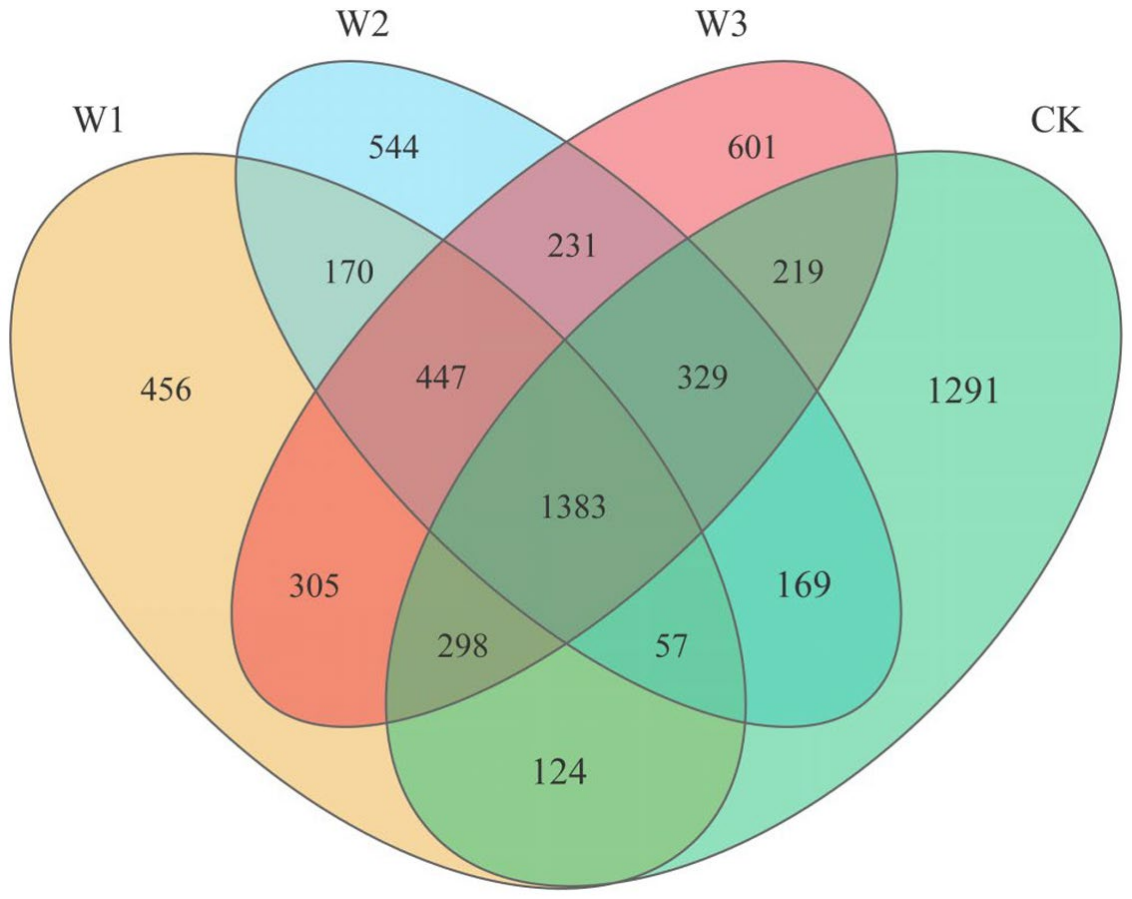
Venn diagram of bacterial OTUs from the soil samples.

After quality filtering, a total of 721,528 bacterial sequences were obtained from 12 samples, which were clustered into 14,253 bacterial OTUs. The Venn diagram shows the quantitative relationship of soil bacteria OTUs in different polluted areas (Figure 4). The total number of OTUs in the W1, W2, W3, and CK groups were 3,240, 3,330, 3,813, and 3,870, respectively. Concurrently, the number of endemic OTUs in the W1, W2, W3, and CK groups were 456, 544, 601, and 1,291, respectively. With the aggravation of pollution, the number of soil bacteria OTUs gradually decreased. The degree of pollution directly decreased the number of soil bacteria. Moreover, there is a certain regularity in the shared OTUs between the polluted areas (W1, W2, W3) and the clear area (CK). The Venn diagram illustrated (Figure 4) that W1, W2, and W3 shared 1862, 1938, and 2,229 OTUs, respectively, compared to the CK group. Additionally, the shared OTUs between W1 and W2, W1 and W3, and W2 and W3 were 2057, 2,431, and 2,390, respectively. These findings indicate that the similarity of soil bacterial communities between polluted and clean areas is largely influenced by the extent of soil pollution. However, the similarity of soil bacterial communities among different polluted areas does not exhibit a clear regularity.

**Figure 5:**
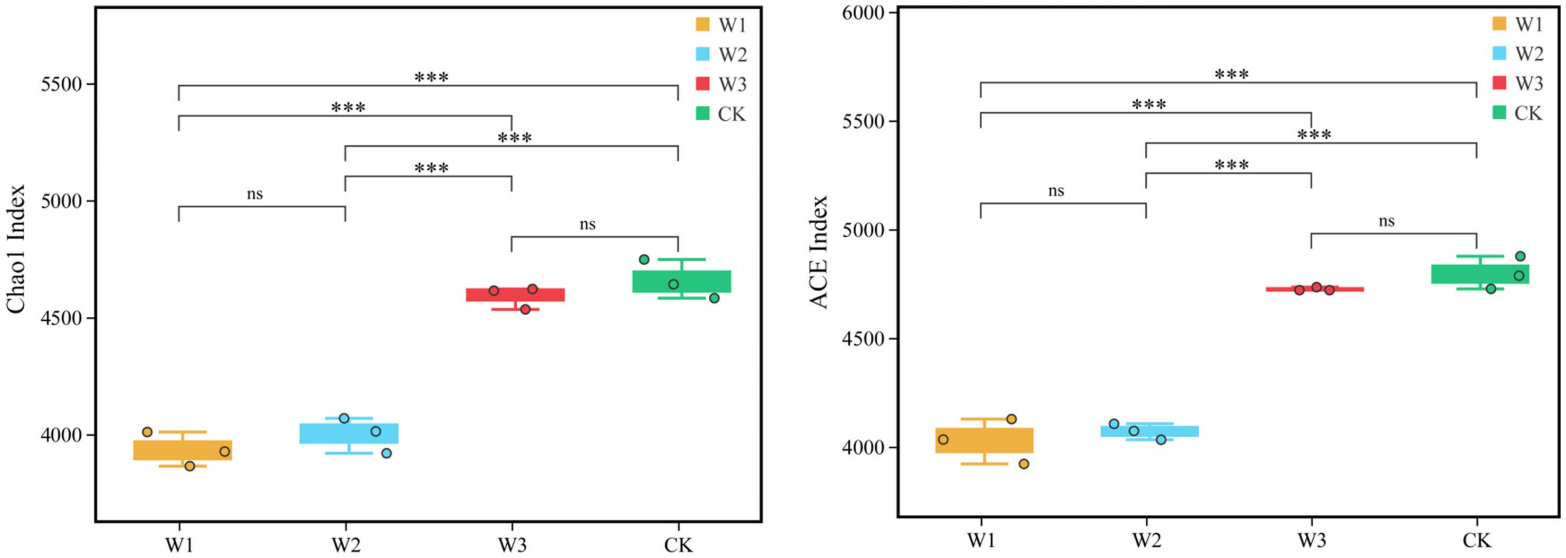
Comparison of the richness of OTUs (Chao1 and ACE indices) in different polluted areas of tailings. *, *p* < 0.05; **, 0.01 < *p* < 0.05; ***; *p* < 0.01; ns, no significance.

Furthermore, Alpha diversity, i.e., the Chao1 and ACE indices represent the richness of OTUs. The ACE and Chao1 indices of W1, W2, W3, and CK groups are compared in Figure 5. The results showed that the ACE and Chao1 indices showed a consistent trend, and the richness of OTUs in the soil increased with decreasing tailings pollution. Compared with CK, the ACE and Chao1 indices of the W1 group significant decreased by 16.03 and 15.54% (*p* < 0.01), and the W2 group significant decreased by 15.14 and 14.11% (*p* < 0.01), respectively. However, the difference in the richness of OTU between W3 and CK was not statistically significant.

### 3.4. Comparison of the sequencing depth of alpha diversity in the soil with different pollution levels

**Figure 6:**
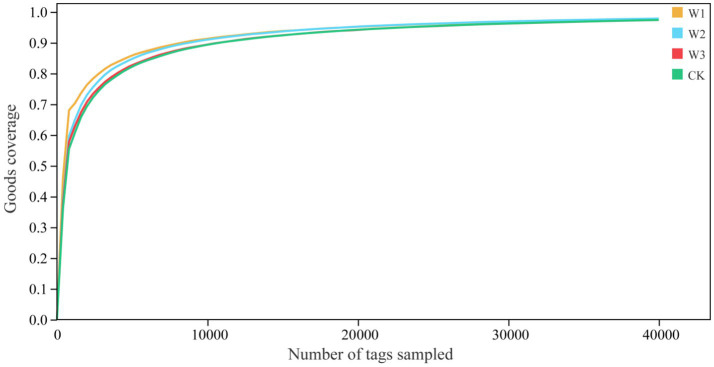
Analysis of the dilution curve of goods coverage indices in different polluted areas and clean area.

**Figure 7:**
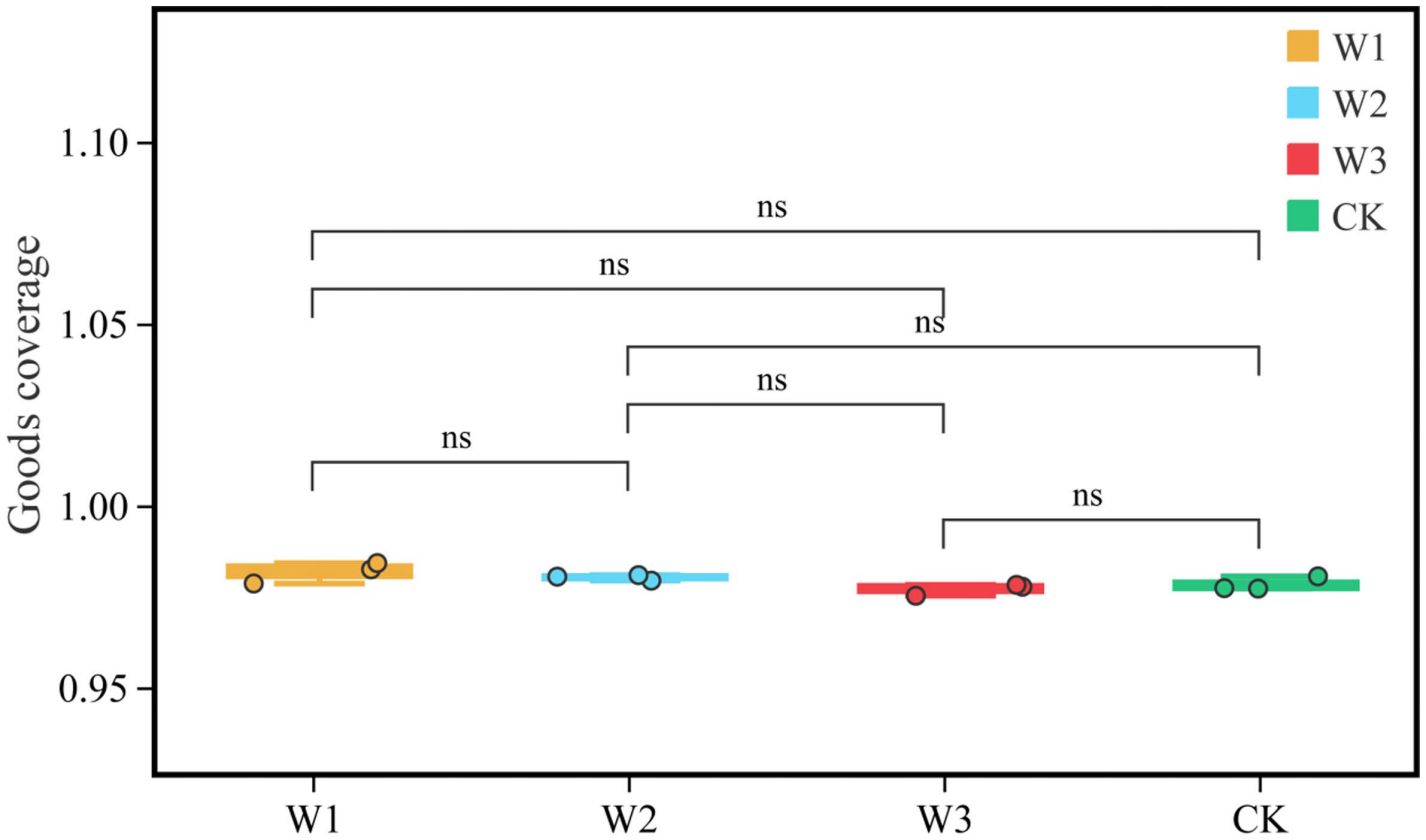
Comparison of goods coverage indices in different polluted areas and clean areas. ns, no significance.

We evaluate whether the sequencing quantity is enough by drawing the dilution curve of the Goods coverage index using QIIME software. The dilution curve results showed that the dilution curve of clean area and different polluted areas tended to be flat finally, it suggests that the increase of sequencing depth had not affected the species diversity, and the sequencing amount was sufficient (Figure 6). Furthermore, it as can be seen from Figure 7, there is no significant difference in the Goods coverage index among different areas.

### 3.5. Comparison of beta diversity of soil bacterial community in the soil with different pollution levels

**Figure 8:**
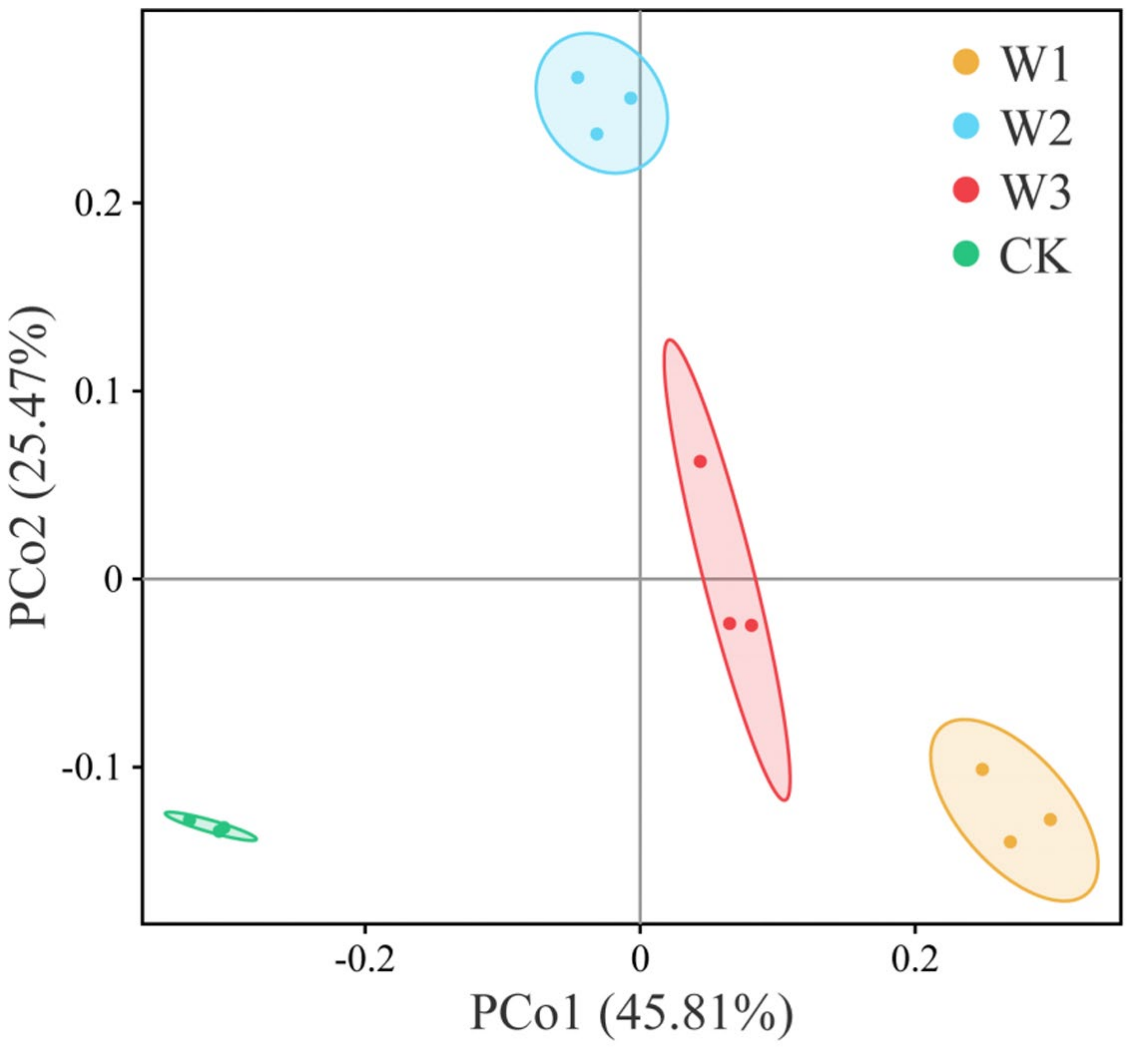
Differences in bacterial community composition under different polluted areas of tailings by principal coordinate analysis (PCoA).

The principal coordinate analysis (PCoA) was performed to evaluate the bacterial community composition across different polluted areas under tailing soils (Figure 8). PCoA results showed that bacterial community under different polluted areas was different, where PCo1 and PCo2 accounted for variations of 25.47 and 45.81%, respectively. Bray-Curtis also showed that bacterial communities under different polluted areas was highly significantly different compare with CK groups (*p*<0.01). Furthermore, we also revealed no significant bacterial community differences for W1 vs. W2 (*p* = 0.412), W1 vs. W3 (*p* = 0.135), and W2 vs. W3 (*p* = 0.1832; Table 3). These results showed that heavy metal contamination lead and zinc significantly affected bacterial community composition for tailings soils, and the influence of bacterial community was related to the degree of pollution.

**Table 3:** Significance test of differences among bacterial communities in different polluted areas using permutational analysis of variance based on Bray-Curtis distance.

Soil source	Groups	Measure	Permmutations	*p* value	Signification
Tailing	CK *VS* W1	Bray	999	0.0080	***
	CK *VS* W2	Bray	999	0.0094	***
	CK *VS* W3	Bray	999	0.0397	**
	W1 *VS* W2	Bray	999	0.4122	ns
	W1 *VS* W3	Bray	999	0.1357	ns
	W2 *VS* W3	Bray	999	0.1832	ns

Star represents significance (*, *P* < 0.05; **, 0.01 < *P* ≤ 0.05, ***, *P* ≤ 0.01; while no star represents no significance).

### 3.6. Analysis of the soil bacterial community structure in different polluted areas

**Figure 9:**
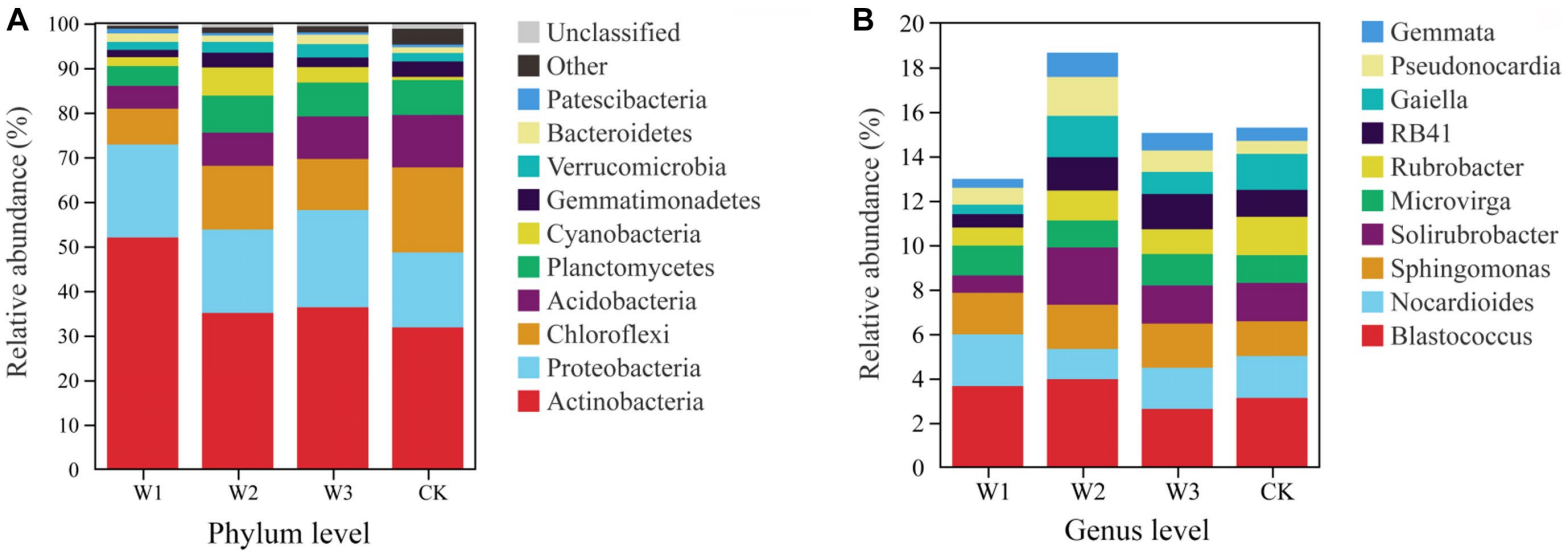
The bacterial community structure at the phylum level **(A)** and genus level **(B)**.

At the phylum level, *Actinobacteria*, *Proteobacteria*, and *Chloroflexi* are the dominant bacteria in different polluted areas and clean areas, accounting for more than 65% of the total number of soil bacteria OUTs in Figure 9A. The relative abundance of dominant bacteria–*Actinobacteria* and *Proteobacteria* in polluted areas of tailings was higher than that in the CK. Besides, the highest relative abundance of *Actinobacteria* in the W1 groups was 51.95%, which increased by 63.41% with CK. On the contrary, the relative abundance of *Chloroflexi* in polluted areas was lower as compared to the one in CK, and the relative abundance of *Chloroflexi* in CK is as high as 19.8%, which is 137.31% higher than that in W1. On the whole, due to the influence of tailings pollution, the top three dominant phylum in soil did not change, but the relative abundance of dominant phylum was significantly affected, among which *Actinobacteria* and *Chloroflexi* were greatly affected. The relative abundance of the top 3 bacterial genera is shown in Figure 9B. With the aggravation of tailings pollution, *Blastococcus*, *Nocardioides*, and *Solirubrobacter* behave irregularly in the four groups. Among them, the highest values of relative abundance of *Blastococcus*, *Nocardioides*, and *Solirubrobacter* appeared in W2, W1, and W2 groups respectively, which increased by 26.92, 23.28, and 50.00%, respectively compared to CK.

**Figure 10:**
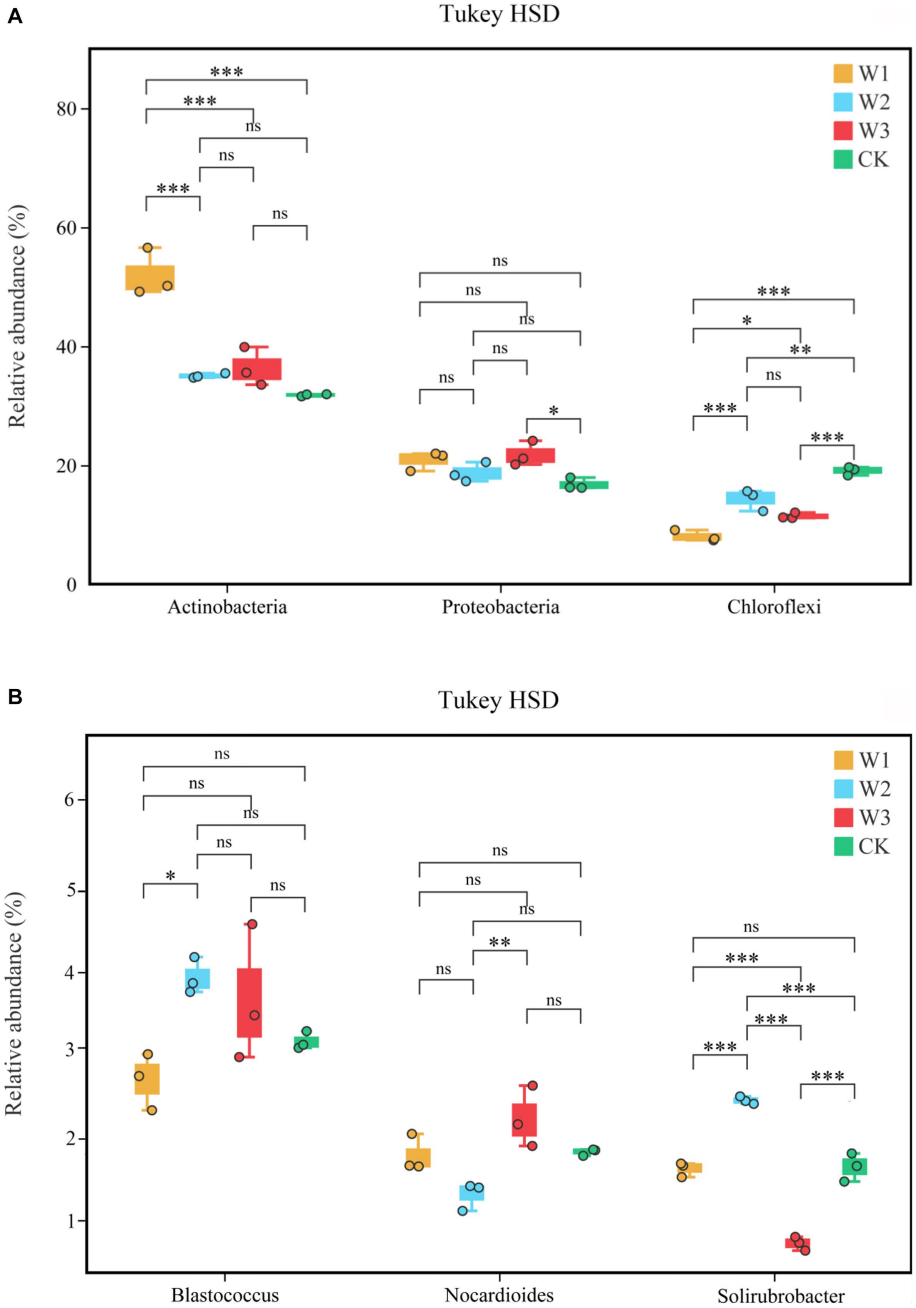
The soil bacterial community structure at the phylum level **(A)** and genus level **(B)**, Tukey HSD test at genus level with 95% confidence intervals. *, *p* < 0.05; **, 0.01 < *p* ≤ 0.05, **, *p* ≤ 0.01; ns, no significance.

The difference in the relative abundances of *Actinobacteria*, *Proteobacteria*, and *Chloroflexi* in different pollution areas by Tukey HSD detection is shown in Figure 10A. Among them, compared with the CK, the relative abundance of *Actinobacteria* increased significantly and that of *Chloroflexus* decreased significantly in the W1 group (*p < 0.01*), but the relative abundance of *Proteobacteria* did not change notably. Compared with the CK, the relative abundance of *Cloroflexures* decreased (*p* < 0.01) and that of *Proteobacteria* increased significantly in the W3 group (*p* < 0.05), but the relative abundance of *Actinobacteria* did not change notably. No significant differences were observed in the relative abundances of *Blastococcus* and *Nocardioides* in different polluted areas compared to the CK group in Figure 10B. In contrast, compared with the CK, the relative abundances of *Solirubrobacter* under the W2 and W3 groups were significantly different, but there was no significant difference in the W1 group.

### 3.7. Constrained relationship between soil’s physical and chemical properties and soil bacterial community

**Figure 11:**
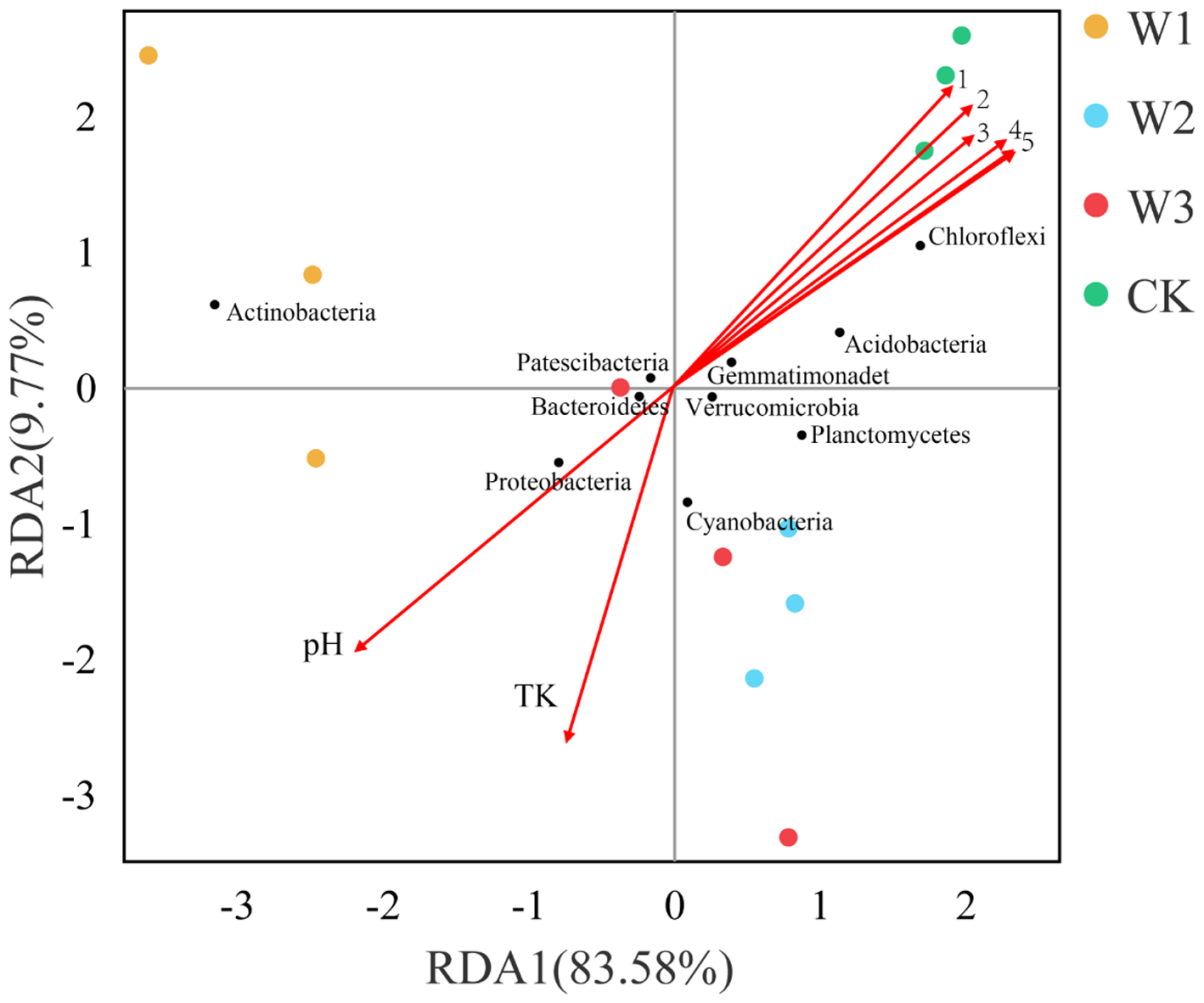
The RDA between soil physical and chemical properties and the soil bacterial communities at the phylum level. 1, AP; 2, AK; 3, TP; 4, AN; 5, TN and OC.

To further analyze the influence of physical and chemical properties of the soil on bacterial community structure in different tailing-polluted areas. The RDA of the soil bacterial community and soil’s physical and chemical properties in shown in Figure 11. The two axes of the RDA explain the total variance of 83.58 and 9.77%, respectively. The combined value of the first two axes explains 93.35% of the total variance of the soil bacterial community structure, indicating the reliability of the RDA analysis. Out of these factors, TN, AN, TP, OP, AK, and OC levels were positively correlated to *Chloroflexi* and negatively correlated to *Actinobacteria* and *Proteobacteria*. The TK and pH levels were positively correlated to *Actinobacteria* and *Proteobacteria*.

**Figure 12:**
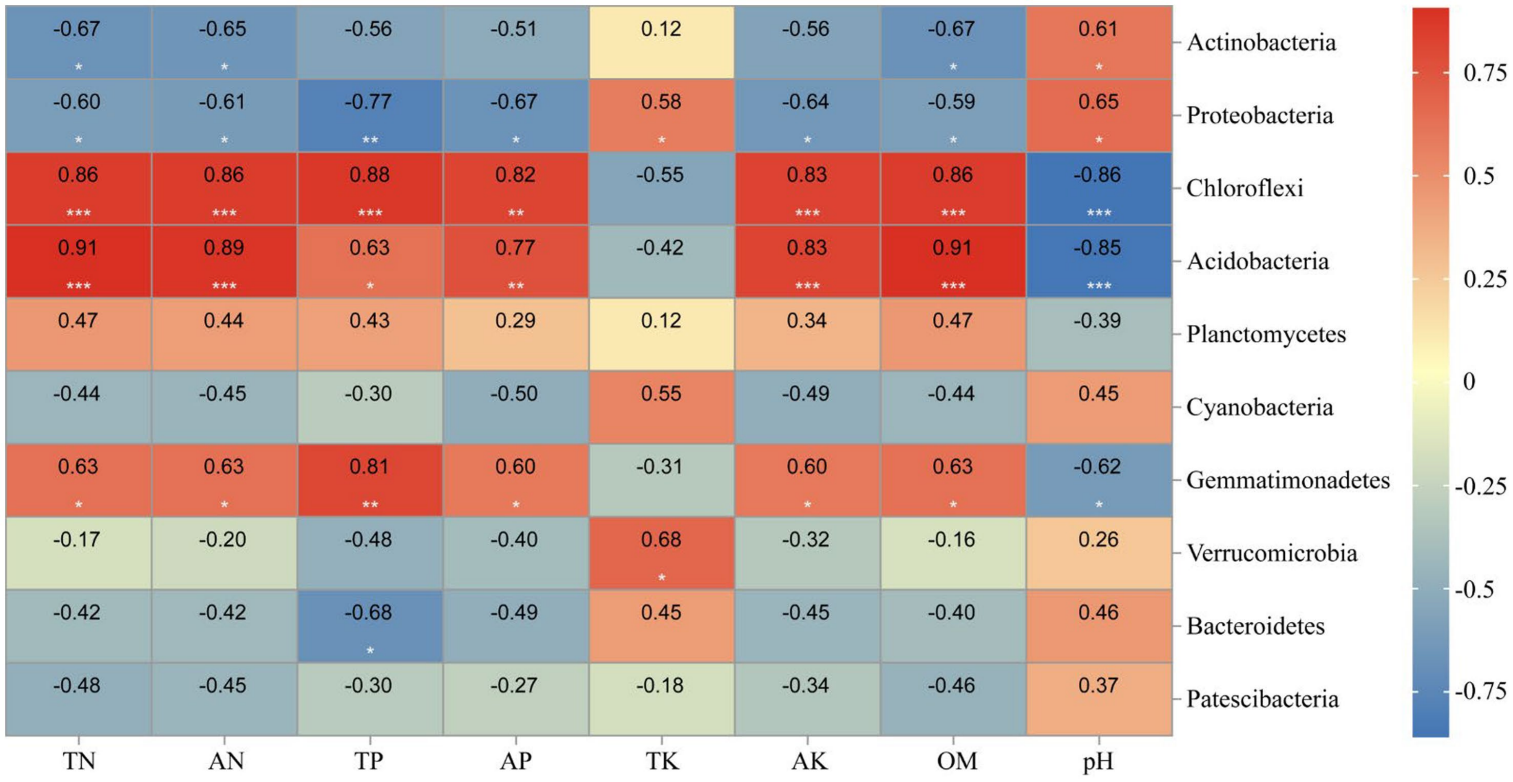
Heatmap showing the strength of correlation between the soil properties and bacterial community. *, *p* < 0.05; **, 0.01 < *p* ≤ 0.05; **, *p* ≤ 0.01. Numerical value represents R value.

Correlation heat map showed that *Actinobacteria* was significantly positively negatively with TN, AN, and OM levels, while positively correlated with soil pH (Figure 12). Besides, all environmental factors could significantly affect *Proteobacteria* (*p*<0.05). TN, AN, TP, AK, and OM were significantly positively correlated to *Chloroflexi* (*p*<0.01), while negatively correlated with pH (*p*<0.01).

### 3.8. Analysis of biological metabolic pathway based on Tax4 Fun

**Figure 13:**
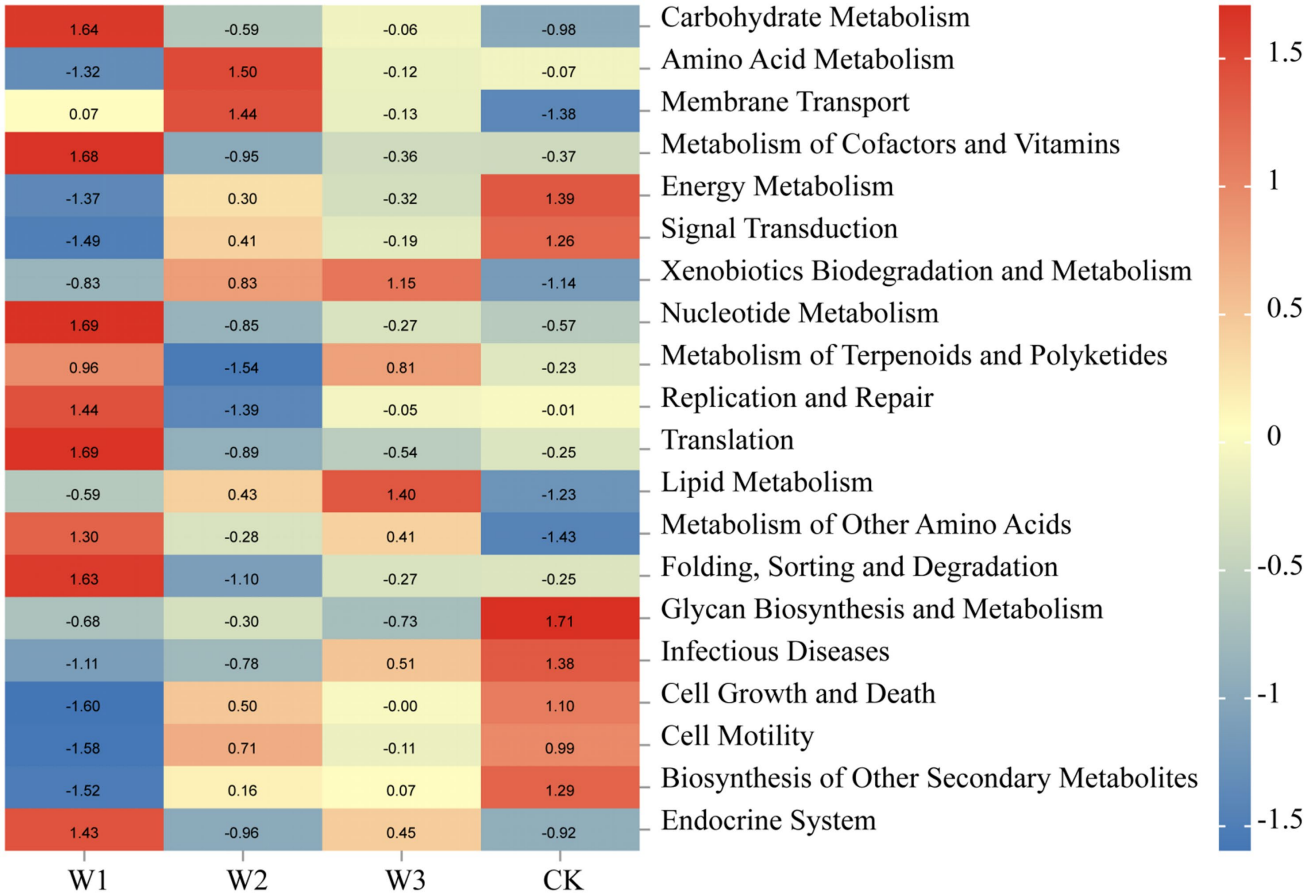
Main metabolic pathways of the soil bacteria in different areas of tailings based on Tax4 Fun. Legend bar and numerical value represent *R* value.

Tax4 Fun was normalized, which analyze the biological metabolic pathways of the soil bacterial communities in different pollution areas of tailings. According to the prediction results of Tax4 Fun in Figure 13, the biological metabolism of the soil bacterial community was significantly altered due to the aggravation of heavy metal pollution. Soil bacterial communities in the W1 were primarily involved in metabolic processes such as carbohydrate metabolism, metabolism of cofactors and vitamins, nuclear metabolism, translation, folding, sorting and degradation, and endocrine system. Amino acid metabolism and membrane transport were the most dominant metabolic processes in the W2. Lipid metabolism was the dominant metabolic process in the W3 whereas the metabolic processes primarily observed in the CK were energy metabolism, glycan biosynthesis, and metabolism. Overall, the metabolic process of the soil bacterial community was affected by the altered soil environment. In areas with different degrees of soil pollution, the primary biological metabolic processes involved in different districts were different. These results further indicate that the soil bacterial communities under the influence of tailings play an important role in the metabolic processes in the tailing-polluted areas.

## 4. Discussion

### 4.1. Effects of the tailing pollution on the physical and chemical properties of the soil

The physical and chemical properties of the soil are the primary indicators of soil nutrients and determine the overall fertility level of the soil. Previous studies have shown that the available nutrients of the soil, such as AN, AP, and AK are easily affected by the environment, resulting in nutrient loss, and their levels directly reflect the soil quality (Zhou et al., 2020). At present, Various results have been reported on the effects of heavy metal pollution degree on soil available nutrients, such as, AN, AP, AK, and OC levels in the soil. Some studies have found that soil available nutrients increased significantly with an increase in heavy metal pollution (Wei et al., 2018; Liu et al., 2022). while others reported that the levels of soil available nutrients could increase under the environment of low levels pollution of heavy metals (Wang et al., 2023). These different results may be related to the types of heavy metals and the degree of heavy metal pollution in soil. In this experiment, the AN, AP, AK, and OC levels value were significantly decreased with the aggravation of pollution in soil, indicating that the soil nutrients were seriously lost and the soil quality deteriorated. These findings consistent with previous findings (Wei et al., 2018; Liu et al., 2022). The accumulation of tailings wastes could be served as an explanation. As it would result in the destruction of the surface vegetation and soil structure, thus reducing the litter at the surface vegetation. In addition, the surface plants were poisoned by the harmful wastes in the tailings, and the degradation rate of the litter was reduced due to the reduced stability of the soil leading to the reduction thus the insufficient levels of soil nutrients (Evans et al., 2012). The perennial accumulation of this kind of hazardous waste from tailings increases the passivation reaction of harmful substances with soil available nutrients, decreases the available nutrients under the soil, and destroys the nutrient balance under the soil. This hampers the growth of surface vegetation. Besides, in this experiment, the contents of available nutrients in soil are positively correlated with lead and zinc pollution areas in different degrees, which represents the concentration of lead and zinc in soil. These findings consistent with previous findings (Liu et al., 2017; Pan et al., 2020).

Soil pH is one of the most critical determinants of soil quality, which is an important factor affecting the content and state of available nutrients in soil (Zhao et al., 2011; Da Silva Cerozi and Fitzsimmons, 2016). In this experiment, Soil pH was increased with the aggravation of pollution, and the concentration of AP was increased with a decrease in soil pH. Because it has been reported that a decrease in pH may result from increasing the activity of proton-coupled solute transporters and enhancing the anion uptakee (White, 2011; Yin et al., 2021). Moreover, it was observed that the pH content of W1 was significantly higher than that of CK. This difference may be attributed to the significant impact of tailings on W1, leading to changes in the degree of surface vegetation damage and the abundance of soil acidic microorganisms, consequently resulting in soil acidification (Evans et al., 2012).

In the present study, we observed that with the aggravation of pollution, soil OC content decreased, and the OC content in the polluted area was significantly lower than CK, which was mainly due to its buffering effect through low nutrient supply and high nutrient storage mechanism, which maintains the carbon and nitrogen cycle in the ecosystem of small tailings areas. However, a significant decrease in the OC level in W1 may be caused by extreme pollution stress, which leads to the imbalance of carbon and nitrogen, and it is difficult to maintain the regional carbon and nitrogen cycle in a short time (Soussana and Lemaire, 2014), which is consistent with the research results of Soussana and Lemaire (2014). The abnormal content of heavy metals in soil not only resulted in different degrees of polluted soil, but also the loss of soil nutrients under polluted conditions may alter the soil microbial abundance and community structure.

### 4.2. Influence of the soil physical and chemical factors on the soil bacterial community structure and metabolic function analysis

In this study, the aggravation of tailings pollution significantly reduced the number of OTUs of the soil microbes (Figure 3) and altered the abundance of soil microbes. On the one hand, it might be due to tailing pollution caused by the altered physical and chemical factors in the soil. We also observed altered levels of nutrients, specifically decreased levels of carbon, nitrogen, and phosphorus required for the growth and metabolism of soil microbes resulting in an intensification of the competition among soil microbes, in line with the study by Zheng et al. (2021). Besides, there are differences between α diversity of the soil bacterial community and CK in different pollution degrees, and the soil bacterial community showed different response results to different pollution areas of tailings. We observed that the ACE and Chao1 indexes were negatively correlated to the pollution degree, i.e., the higher the pollution degree, the lower the richness and diversity of the soil bacteria. On the one hand, the reason may be that the heterogeneity of soil bacterial microenvironment in clean areas is higher, which makes the diversity of soil bacteria higher; On the other hand, due to the aggravation of tailings pollution, the surface native vegetation gradually disappears and the structure of vegetation community tend to be single, and the humus and root exudates transported to the soil will be correspondingly reduced (Paredes and Lebeis, 2016), which is consistent with the previous research results (Luo et al., 2023).

Previous studies have shown that species within these phyla, including (*Acidobacteria*, *Actinobacteria*, *Bacteroidetes*, *Chloroflexi*, *Planctomycetes*, *Proteobacteria*, and *Verrucomicrobia*), have been identified as dominating in heavy metal contaminated soils due to either inherent or acquired tolerances and resistances to toxic metals (El Baz et al., 2015; Kielak et al., 2016; Shen et al., 2016; Gu et al., 2017). In the present study, *Acidobacteria*, *Proteobacteria* and *Chloroflexi* were dominant species in abandoned Zn and Pb tailings areas, but its relative abundance has changed significantly. it might be caused by the different adaptability of different species in response to heavy metal pollution stress (Zhang et al., 2018; Lu et al., 2020). Moreover, some studies showed that the long-term adaptation of soil microbes in the polluted environment resulted in the abundance of individual microbes was higher than that in the clean areas (Avidano et al., 2005). In the present study, compared to CK, the abundance of Actinobacteria in the soil gradually increased to 63.41% with increasing pollution degree, while the aggravation of tailings pollution significantly decreased the number of OTUs of the soil microbes. This indicated that external pollution stress might aggravate the interspecific competition between the soil microbes, resulting in the restriction of the low tolerances microbes, while the emergence of the dominant bacterial, such as *Actinobacteria*. Also, remediation of these soils might be possible using a strain of *Actinobacteria*.

Microbes participating in the process of soil nutrient transformations are often closely connected to their dominant biological metabolic functions (Castaeda and Barbosa, 2017; Chen et al., 2019; Srour et al., 2020). In this study, *Actinobacteria*, *Proteobacteria*, and *Chloroflexi* were identified as the dominant bacterial phyla in the soil. Notably, the relative abundance of *Actinobacteria* in W1 showed a significant difference compared to CK, with an increase from 31.78 to 51.95% in W1. Previous studies have shown that heavy metal pollution stress inhibits the abundance bacteria of organic acid-secreting, thereby affecting soil pH (Evans et al., 2012). Furthermore, the results of RDA in this study revealed a positive correlation between soil pH and *Actinobacteria*, as well as *Proteobacteria*, under the influence of heavy metal pollution stress (W1), which also confirmed the fact that soil pH might indirectly affect the abundance of *Actinobacteria* and *Proteobacteria* under the influence of bacterial communities secreting organic acids. These results are consistent with Zhang et al. (2021) research. In the W1 group, the soil bacterial communities primarily participated in different biological metabolic functions, such as carbohydrate metabolism, metabolism of cofactors and vitamins, nucleotide metabolism, translation, folding, sorting and degradation, and endocrine system. Whereas *Actinobacteria* and *Proteobacteria* emerged as the dominant phylum in the W1 group, which might be participation in these biological metabolic functions. Besides, compared with the clean area, the relative abundance of *Chloroflexi* was significantly decreased in the W1 group. and abundance of energy metabolism, signal transduction, cell growth and death, cell motility and biosynthesis of other secondary metabolites significantly decreased in the W1 group, while these functions are the opposite in CK. these results showed that these biological metabolic functions may be related to the relative abundance of *Chloroflexi* in soil.

The above results indicate that *Actinobacteria* and *Proteobacteria* are the dominant phyla in the tailing-polluted areas and their ecological functions are significantly difference with clean area. To sum up, the change of environmental factors affects the community structure of soil bacteria, which leads to the change of biological metabolism function in which soil bacteria participate. It is worth mentioning that although the exact environmental factors and target bacteria involved in biological metabolic function were not clearly analyzed in this study, it provided effective data for the subsequent research on biological metabolic function.

## 5. Conclusion

In this study, we observed that heavy metal pollution resulted in the loss of soil nutrients. At the same time, based on the results of the Illumina sequencing technology, we concluded that with the aggravation of heavy metal pollution of soil, the richness of soil bacteria OTUs decreased. The richness of OTUs (Chao1 and ACE indices) was also significantly decreased in the heavily polluted area (W1). The relative abundances of *Actinobacteria* and *Proteobacteria* were increased in the polluted areas (W1-W3). The relative abundances of *Actinobacteria* and *Proteobacteria* were increased while the relative abundances of *Chloroflexi* decreased in the W1 group significantly. The physical and chemical properties of the soil explained 93.35% of changes in the soil bacterial community of which abundance of *Actinobacteria* and *Proteobacteria* were primarily affected by the TK content and pH of the soil, while TN, AN, TP, AK, and OM contents of the soil were the key environmental factors to increase abundance of *Chloroflexi*. Soil heavy metal pollution affects the soil bacterial community, influencing the related metabolic processes. This study explained the altered soil bacterial community structure and its primary driving factors in different polluted areas of tailings, revealing the altered ecological functions of the soil bacterial community under heavy-metal stress. These findings provide valuable information for the rational utilization and management of tailings. In view of the feasibility of phytoremediation on heavy metal contaminated soil, future research should focus more on implications and mechanism of phytoremediation on soil nutrients and soil microbes.

## Data availability statement

The datasets presented in this study can be found in online repositories. The names of the repository/repositories and accession number(s) can be found in the article/supplementary material.

## Author contributions

JZ enabled and supervised this research and conceived of the study. ZL designed the study. ZL, CL, and KZ performed the experiments. ZL conducted data analysis and wrote the article. All authors have read and agreed to the published version of the manuscript.

## Funding

This work was funded by the National Key R&D Program of China [2017YFC0505500&2019JSJG247]. The authors declare that they have no known competing financial interests or personal relationships that could have appeared to influence the work reported in this paper.

## Conflict of interest

The authors declare that the research was conducted in the absence of any commercial or financial relationships that could be construed as a potential conflict of interest.

## Publisher’s note

All claims expressed in this article are solely those of the authors and do not necessarily represent those of their affiliated organizations, or those of the publisher, the editors and the reviewers. Any product that may be evaluated in this article, or claim that may be made by its manufacturer, is not guaranteed or endorsed by the publisher.

## Footnotes

^1^ http://www.omicsmart.com
